# Atypical X-linked agammaglobulinaemia caused by a novel BTK mutation in a selective immunoglobulin M deficiency patient

**DOI:** 10.1186/1471-2431-13-150

**Published:** 2013-09-27

**Authors:** Lee-Moay Lim, Jer-Ming Chang, I-Fang Wang, Wei-Chiao Chang, Daw-Yang Hwang, Hung-Chun Chen

**Affiliations:** 1 Division of Nephrology, Department of Internal Medicine, Kaohsiung Medical University Hospital, 100 Tze-You First Road, Kaohsiung City 807, Taiwan; 2 Department of Internal Medicine, Kaohsiung Municipal Hsiao-Kang Hospital, Kaohsiung, Taiwan; 3 Department of Pediatrics, Kaohsiung Medical University Hospital, Kaohsiung Medical University, Kaohsiung, Taiwan; 4 Department of Clinical Pharmacy, School of Pharmacy Taipei Medical University, Taipei, Taiwan; 5 Master Program for Clinical Pharmacogenomics and Pharmacoproteomics, School of Pharmacy, Taipei Medical University, Taipei, Taiwan; 6 Faculty of Renal Care, College of Medicine, Kaohsiung Medical University, Kaohsiung, Taiwan

**Keywords:** X-linked agammaglobulinaemia, Bruton’s tyrosine kinase, Proteinuria, Haematuria, Immunoglobulin

## Abstract

**Background:**

X-linked agammaglobulinaemia (XLA) is the most common inherited humoural immunodeficiency disorder. Mutations in the gene coding for Bruton’s tyrosine kinase (BTK) have been identified as the cause of XLA. Most affected patients exhibit a marked reduction of serum immunoglobulins, mature B cells, and an increased susceptibility to recurrent bacterial infections. However, the diagnosis of XLA can be a challenge in certain patients who have near-normal levels of serum immunoglobulin. Furthermore, reports on XLA with renal involvement are scant.

**Case presentation:**

We report an atypical XLA patient who presented with selective immunoglobulin M (IgM) immunodeficiency and nephropathy. He was diagnosed with selective IgM immunodeficiency, based on his normal serum immunoglobulin G (IgG) and immunoglobulin A (IgA) levels but undetectable serum IgM level. Intravenous immunoglobulin was initiated due to increased infections and persistent proteinuria but no improvement in proteinuria was found. A lupus-like nephritis was detected in his kidney biopsy and the proteinuria subsided after receiving a mycophenolate mofetil regimen. Although he had a history of recurrent bacterial infections since childhood, XLA was not diagnosed until B-lymphocyte surface antigen studies and a genetic analysis were conducted.

**Conclusions:**

We suggest that B-lymphocyte surface antigen studies and a BTK mutation analysis should be performed in familial patients with selective IgM deficiency to rule out atypical XLA.

## Background

X-linked agammaglobulinaemia (XLA) (OMIM # 300755) is a humoural immunodeficiency disease characterised by severe hypogammaglobulinaemia, defective B cell development, and extremely decreased numbers of mature B cells [1]. In 1952, Colonel Ogden Bruton described the first case of XLA in a boy with a history of recurrent bacterial infections [2]. The gene responsible for XLA was identified in 1993 and named Bruton’s tyrosin kinase (BTK) [3]. The *BTK* gene is localised at Xq21.3-Xq22 and contains 19 exons spanning 37.5 kb [4]. A member of the Tec family, the *BTK* gene is a cytoplasmic tyrosine kinase that plays a critical role in the development of B cells [5]. Five domains of BTK, comprising pleckstrin homology (PH), Tec homology (TH), Src homology 3 (SH3), Src homology 2 (SH2), and the kinase domain TK, have been identified, with each having a distinctive function [5]. The lack of functional BTK results in defective B cell development at the pro-B and pre-B cell stages [6], leading to a reduction of mature B cells in the peripheral blood. The clinical diagnosis of XLA depends on a positive family history of immunodeficiency, recurrent bacterial infections before the age of 5 years, life-threatening bacterial infections in early childhood, and considerably low levels of all isotypes of serum immunoglobulins [7]. These indications are necessary for a definite diagnosis of XLA: the patient must be male and have less than 2% CD19+ B cells with mutations in the *BTK* gene, absent BTK mRNA on a northern blot analysis of neutrophils or monocytes, absent BTK proteins in monocytes or platelets, as well as maternal cousins, uncles, or nephews who have mutations [8].

Most XLA-afflicted boys were diagnosed with repeated or protracted bacterial infections during early childhood after their maternal immunoglobulins had been lost [9], and before the era of the intravenous immunoglobulin (IVIG) and antibiotics, the disease could be life threatening. Currently, only 2 XLA cases associated with nephropathies can be found in the literature [10,11]. Here, we report an atypical XLA case occurring with a novel *BTK* mutation in a Chinese boy presenting with nephritis and selective IgM deficiency.

## Case presentation

A 6-year-old Chinese boy with a 2-year history of persistent haematuria and proteinuria found by routine screen was referred to our department. He had suffered several episodes of otitis media and maxillary sinusitis since the age of 3 years without requiring hospitalisation. He was diagnosed with selective IgM deficiency at the age of 5 years. Clinical examinations revealed a normal gross appearance and growth percentile, and there was no pitting edema or skin rash. His family history was unremarkable except that his elder brother, who had experienced recurrent sinusitis and atopic dermatitis, had been diagnosed with selective IgM deficiency at the age of 3 years. His brother had received intravenous immunoglobulin (IVIG) treatments and has normal renal function without proteinuria and haematuria. Examining our patient’s kidneys by using ultrasound revealed that his kidneys and urinary tract system were grossly normal. Performing a dipstick urinalysis revealed that the urine contained occult blood 3+ and protein 2+. His daily protein loss was 1.4 g/d. Other blood and urine biochemistry data, including titres of the antinuclear antibodies, antistreptolysin-O, and autoantibodies related to systemic lupus erythematosus were all negative (Table 1).

**Table 1:** Clinical characteristics of our patients with X-linked agammaglobulinemia

	**Index case**	**Brother**	**Reference range**
**Age years**	6	8	
**WBC × 1000/ul**	6.5	6.7	6.0-10.4
**Seg %**	58	38	27.8-57.6
**Lymph %**	36	47	34.4-62.8
**Mono %**	3	8	2.0-7.6
**Eosinophil %**	1	5	0-6.8
**IgG mg/dl**	823	963	608-1572
**IgA mg/dl**	129	267	33-236
**IgM mg/dl**	<4.17	11.1	48-242
**IgE IU/ml**	184	2160	Child (3–9 y/o) : < 53 IU/mL
**C3 mg/dl**	48.2	66.9	77-195
**C4 mg/dl**	12.5	16.5	7-40
**CH50 CAE unit**	46.5	71.97	63-145
**ANA**	1:40	<1:40	
**Anti-dsDNA IU/ml**	0.7		<10, negative
**Anti-Ro U/ml**	3.2		<7, negative
**Anti-La U/ml**	0.3		<7, negative
**Anti-Sm U/ml**	0.1		<5, negative
**Anti-nRNP U/ml**	0.8		<5, negative
**Antistreptolysin-O IU/ml**	<25.0		<100 IU
**Urine protein**	2+	-	Negative
**Urine OB**	3+	-	Negative
**Serum Creatinine mg/dl**	0.4	0.42	0.2-1.0
**CD3 %**	82	94.8	Child (>2 y/o) : 58-87
**DR+/CD3 + %**	12	15.5	
**CD19 %**	1	0.8	Child (>2 y/o) : 5-23
**CD4 %**	44	35.9	Child (>2 y/o) : 32-62
**T-cell subsets CD8 %**	37	49.3	Child (>2 y/o) : 12-45
**HLA-DR positive %**	18	16.8	

**Figure 1:**
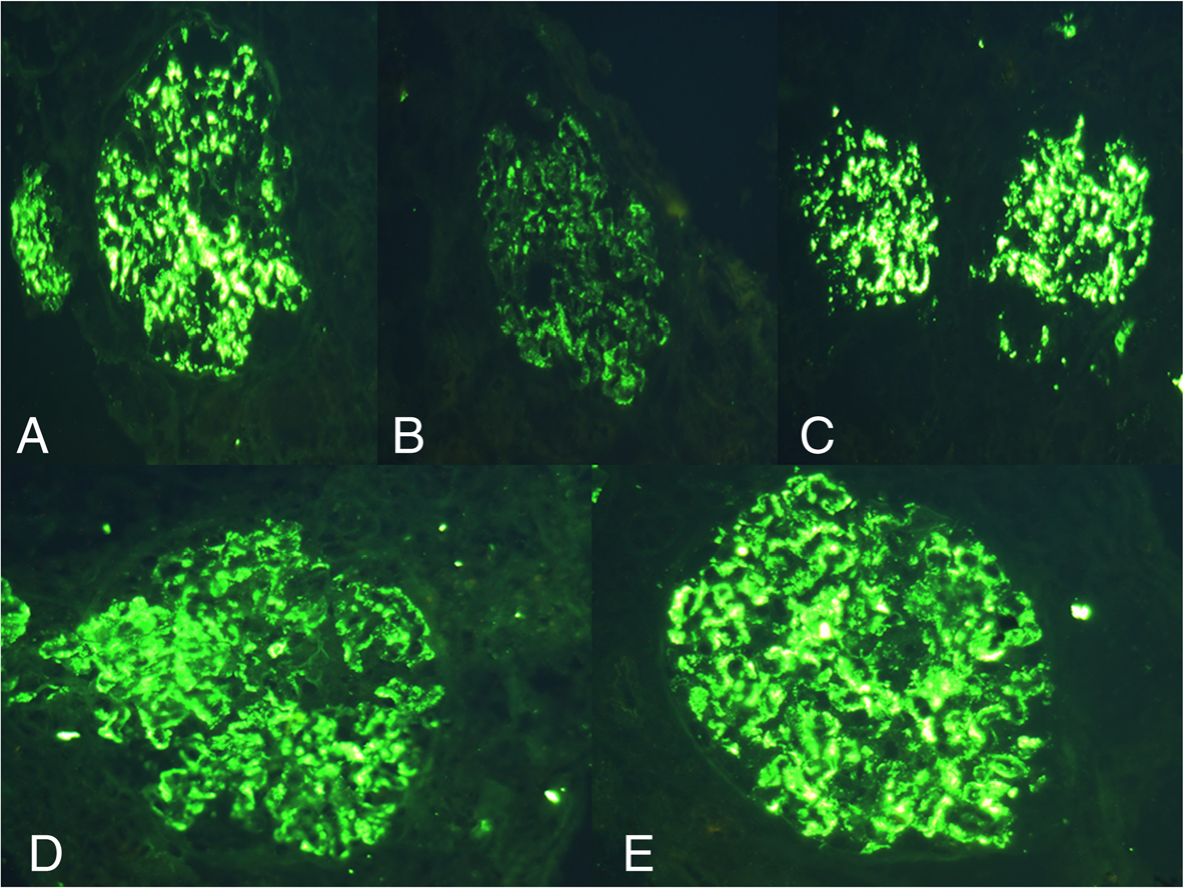
Immunofluorescence microscopy showed strong staining of (A) IgG, (B) IgA, (C) C3, (D) IgG kappa, and (E) IgG lambda over mesangium and glomerular basement membrane (original magnification, × 400).

**Figure 2:**
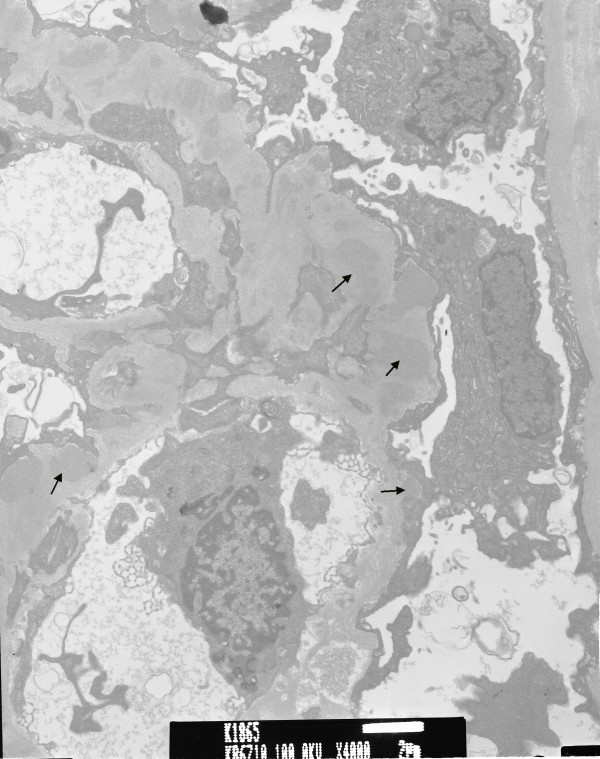
Electron microscopy revealed diffuse foot process effacement and electronic dense deposition (arrows) over subendothelial, subepithelial, and paramesangial areas (original magnification, × 4,000).

**Figure 3:**
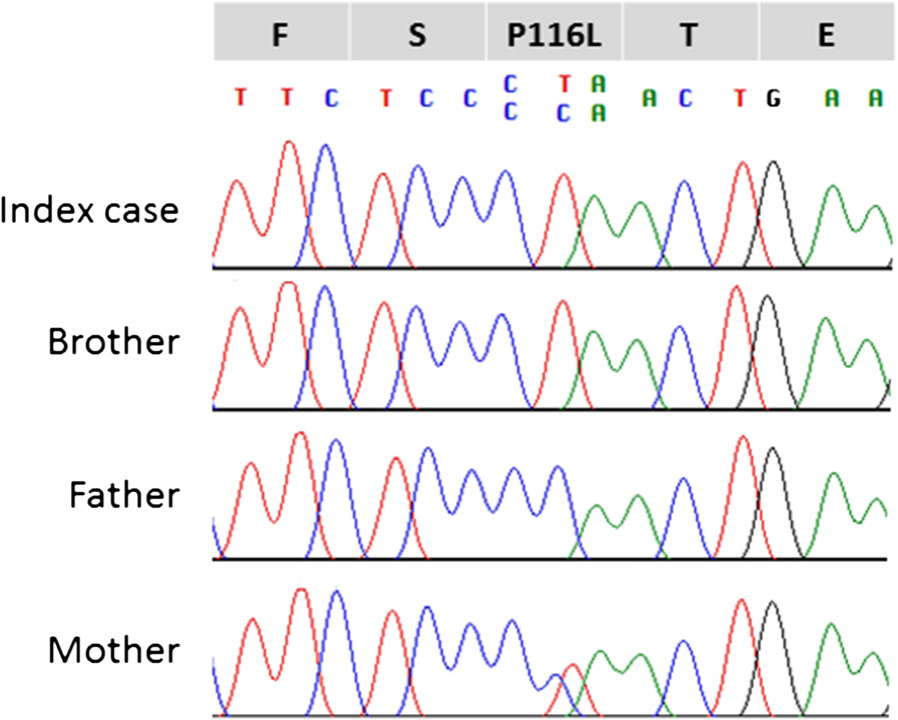
Chromatogram sequence of index patient and family members.

At the age of 6 years, the patient received 20 mg/d of prednisolone orally for 3 months, which was later combined with 2 mg/d of chlorambucil for a further 6 months. Neither treatment improved his proteinuria and haematuria. He suffered from more frequent episodes of sinusitis during this treatment. Because of increased episode of infections and persistent proteinuria, the treatment regimen was followed by an IVIG of 400 mg/kg/4 wk for a total of 16 weeks with no change in his proteinuria. Three months after the first IVIG therapy, he was referred to us because of the proteinuria, and a renal biopsy was performed. Under light microscopy, only a mild increase in the glomerular cellularity was noted. Immunofluorescence microscopy demonstrated a strong staining of IgG, IgA, C3, IgG κ, and λ in the mesangium and glomerular basement membrane with equivocal patterns of IgM and C1q (Figure 1A-E). Electron microscopy revealed diffuse foot process effacement and electronic dense deposits over the subendothelial, subepithelial, and paramesangial areas, where focal proliferative lupus nephritis was suspected (WHO Class III) (Figure 2). These lupus-like pathology results were inconsistent with his clinical and autoimmune profile, whereby the diagnosis of systemic lupus erythematosus cannot be made. His following treatment regimen for nephritis consisted of 10 mg/d of prednisolone orally, in addition to a 6-month regimen of 500 mg/m^2^/d of mycophenolate mofetil. His proteinuria and haematuria gradually improved. Further analysis of the surface antigens of T cells and B cells showed considerably low CD19+ (Table 1), where XLA was one of the potential diagnoses. An analysis of the *BTK* gene revealed that the patient and his brother both exhibited a c.347C > T (p.P116L) mutation inherited from their mother (Figure 3). After a 2-year follow up, our patient remains proteinuria-free with normal kidney function and no infections. Written informed consent was obtained for the subjects included in this study and was approved by the Kaohsiung Medical University Hospital Institutional Review Board. The reference sequences for the *BTK* gene are NG_009616.1 and NM_000061.2.

## Discussion and conclusions

Eighty-five percent of patients with an early onset of infections, pan-hypogammaglobulinaemia, and markedly reduced B cells have BTK mutations [12]. Besides *BTK*, mutations in the genes involved in pre-B cell receptor and B cell receptor signalling, including *IGHM* (μ heavy chain), *IGLL1* (λ5), *CD79A* (Igα), *CD79B* (Igβ), *BLNK*, and *LRRC8A*, were reported to block B cell development and cause a similar clinical phenotype [13-18]. Recently, a patient with an absence of p85α, resulting in an early and severe defect in B cell development accompanied with agammaglobulinaemia, but with minimal findings in other organ systems was reported [19].

Amino acid P116 is well conserved among different species. The variant c.347C > T was not observed in the BTK base (http://bioinf.uta.fi/BTKbase/), HGMD Biobase (http://www.biobase-international.com/product/hgmd), 1000 Genomes Project (http://www.1000genomes.org/) or the NHLBI Exome Sequencing Project (ESP) (http://evs.gs.washington.edu/EVS/). The direct sequencing of 100 people of Chinese origin from Taiwan was performed and the c.347C > T variant was not detected (data not shown). Computer estimations of the function of p.P116L are labelled “disease-causing” according to MutationTaster (http://www.mutationtaster.org), and “probably damaging” (a HumDiv score of 0.998 and a HumVar score of 0.949) according to PolyPhen-2 (http://genetics.bwh.harvard.edu/pph2/). Further analyses, including the expression of BTK in mononuclear cells from the patients and the alteration in the tyrosine 223 phosphorylation in monocytes after activation through Toll-like receptors [20], should provide the degree of functional defect of this mutation.

Up to 10% of XLA patients have atypical presentation and exhibit normal or near normal serum immunoglobulin levels or show significant levels of serum IgG (approximately 800 mg/dL) with variable clinical findings [21]. They are typically diagnosed at an older age with less severe phenotypes. The CD19+ B cell remains less than 1%, but certain “leaky” B-cells mature with higher immunoglobulin levels in these atypical XLA cases [21]. The mechanism of this “leaky” phenomenon remains unknown. Mutations that cause atypical XLA are similar to those that cause classic XLA and include single amino acid substitutions, splicing defects, premature stop codons, promoter defects, and gene rearrangement [22]. Normal levels of IgG accompanied with decreased IgM have been reported in other cases involving BTK mutation in XLA [23], but no autoimmune diseases have been reported. The genotype-phenotype association in XLA has been studied, but a strong correlation has not been established [24,25]. It is believed that other genetic and environmental factors might affect the diverse phenotypes of XLA. The delay in diagnosis is typically because of the significant levels of serum immunoglobulins regardless of the severity of clinical phenotypes [26], as in our patient. The low serum IgM led to the diagnosis of IgM deficiency in our patient and his brother before further analyses could be performed. The accurate diagnosis of XLA occasionally requires *BTK* mutational analysis that identifies mutations, which may reside in any domain of the gene [27].

Up to 15% of XLA patients may present different autoimmune manifestations including arthritis, diabetes, hemolytic anaemia, scleroderma, and alopecia [28,29]. Autoimmunity accompanied by kidney disease is rarely reported in XLA cases. There are 2 cases in the literature describing the IVIG-related nephropathies in XLA [10,11]. Yoshino *et al.* reported a 3-year-old boy with XLA who suffered from nephrotic syndrome with the diagnosis of membranoproliferative glomerulonephritis [10]. In this case, various IVIG preparations did not improve the proteinuria and haematuria until methylprednisolone pulse therapy was introduced. Endo *et al.* described a 23-year-old European man who had considerably mild proteinuria with a diagnosis of XLA [11]. The patient received several IVIG courses and developed idiopathic membranous glomerulopathy during follow-up. IVIG treatment was considered the cause of immune depositions in the kidneys of these 2 patients. Our patient developed proteinuria and haematuria before IVIG treatments, and the renal biopsy performed shortly after IVIG treatment showed diffuse immune complex depositions. Compared with the patients in the previous 2 cases who received several years of IVIG supplements, our patient received a relatively small amount of IVIG. The kidney immune complex depositions either developed quickly after IVIG without additional kidney damage (regarding proteinuria level), or these depositions actually occurred before IVIG was administered. Although the origin of the immune complex depositions remained unknown without a kidney biopsy before IVIG supplements, our case raised the possibility that nephropathy can be an entity of autoimmune diseases in XLA.

The goal of IVIG therapy in XLA is to maintain serum IgG levels at 500–800 mg/dL and prevent recurrent bacterial infections that could be life threatening [30,31]. However, the treatment of autoimmunity in XLA is less documented. In many cases, increasing the IVIG dosage may ameliorate the autoimmune phenomenon [32]. The nephropathy in our patient showed a resistance to steroids, chlorambucil, and IVIG treatment (although the dose is less than the typical dose for autoimmune disease), but it responded well to mycophenolate and the patient remained disease free.

Selective IgM deficiency is a rare form of dysgammaglobulinaemia with an incidence of less than 0.003% in the general population [33,34]. The clinical and laboratory criteria are poorly defined, either by an IgM level less than 2 standard deviations below standard, less than 10% of age-adjusted normal controls, or absolute levels less than 10–20 mg/dL [35]. Immunoglobulin M deficiency can be presented as a primary or secondary disease. Secondary diseases are more commonly seen and are often reported to be associated with bacterial and viral infections, autoimmune diseases, and malignancies [36,37]. Currently, no molecular defect has been determined to be responsible for IgM deficiency, and IVIG may be instituted in cases of recurrent, debilitating, or life-threatening infection, and/or in patients with concomitant functional IgG deficiencies [36]. Two patients in an IgM deficiency study exhibited equal to or less than 2% CD19+ B cells [38], indicating that XLA should be a differential diagnosis in familial selective IgM deficiency despite normal IgG levels, as in our patient.

In summary, we reported a family with 2 siblings whose immunoglobulin profiles, except their low CD19+ B cell levels, were similar to selective IgM immunodeficiency. A genetic analysis of this family revealed a novel *BTK* variant, p.P116L, in both siblings, which makes XLA the probable diagnosis. We therefore suggest that an analysis of the B-lymphocyte surface markers and *BTK* gene should be performed in familial patients diagnosed with selective IgM deficiency, which can be an atypical presentation of XLA. Furthermore, we reported another rare glomerulonephritis case and demonstrated the successful treatment of nephropathy in XLA-related autoimmunity by using mycophenolate.

### Consent

Written informed consent was obtained from the mother of the patient for publication of this case report and any accompanying images. A copy of the written consent is available for review by the Series Editor of this journal.

## Abbreviations

XLA: X-linked agammaglobulinemia; BTK: Bruton’s tyrosine kinase, PH, Pleckstrin homology; TH: Tec homology; SH2: Src homology 2, SH3, Src homology 3; IVIG: Intravenous immunoglobulin

## Competing interests

The authors declare that they have no competing interests.

## Authors’ contribution

DYH and LML designed and performed research, analysed and interpreted data, and wrote the manuscript; HCC analysed and interpreted data, wrote the manuscript. JMC, WCC and IFW analysed and interpreted data. All authors read and approved the final manuscript.

## Pre-publication history

The pre-publication history for this paper can be accessed here:

http://www.biomedcentral.com/1471-2431/13/150/prepub
